# MassCode Liquid Arrays as a Tool for Multiplexed High-Throughput Genetic Profiling

**DOI:** 10.1371/journal.pone.0018967

**Published:** 2011-04-22

**Authors:** Gregory S. Richmond, Htet Khine, Tina T. Zhou, Daniel E. Ryan, Tony Brand, Mary T. McBride, Kevin Killeen

**Affiliations:** Agilent Technologies, Santa Clara, California, United States of America; Cinvestav, Mexico

## Abstract

Multiplexed detection assays that analyze a modest number of nucleic acid targets over large sample sets are emerging as the preferred testing approach in such applications as routine pathogen typing, outbreak monitoring, and diagnostics. However, very few DNA testing platforms have proven to offer a solution for mid-plexed analysis that is high-throughput, sensitive, and with a low cost per test. In this work, an enhanced genotyping method based on MassCode technology was devised and integrated as part of a high-throughput mid-plexing analytical system that facilitates robust qualitative differential detection of DNA targets. Samples are first analyzed using MassCode PCR (MC-PCR) performed with an array of primer sets encoded with unique mass tags. Lambda exonuclease and an array of MassCode probes are then contacted with MC-PCR products for further interrogation and target sequences are specifically identified. Primer and probe hybridizations occur in homogeneous solution, a clear advantage over micro- or nanoparticle suspension arrays. The two cognate tags coupled to resultant MassCode hybrids are detected in an automated process using a benchtop single quadrupole mass spectrometer. The prospective value of using MassCode probe arrays for multiplexed bioanalysis was demonstrated after developing a 14plex proof of concept assay designed to subtype a select panel of *Salmonella enterica* serogroups and serovars. This MassCode system is very flexible and test panels can be customized to include more, less, or different markers.

## Introduction

Multiplex analytical systems enable parallel detection of biomolecules in one assay and can provide rapid characterization of a sample while saving on cost and resources. Planar arrays (e.g. DNA microarrays), for example, exploit spatial encoding of probes to carry out massively parallel analysis [1]. Also, particle suspension arrays based on various types of encoding technologies have been relatively recently developed for suggested use in high density multiplexed assays [2], [3]. Particle suspension arrays are made of nano- or microparticle solids of various materials that are conjugated with probes that bind specific targets. The individual particles are encoded by a variety of methods, some commercialized, that create unique optical/spectral codes [4], [5], graphical/patterned codes [6], [7], shaped particles [8], composition codes [9], and others [10]. Assays based on particulate arrays can distinguish from thousands to millions of biomolecules in theory, but most have only demonstrated less than 10plex [2], [3]. Solid support-based arrays have one or more of the following disadvantages: microscopy readout (majority), complex manufacturing, high cost, variation in encoding fidelity among particles with the same code in the same lot (e.g. dye intensity), lack of error correction methods, need for particle orientation for detection, low or unknown sensitivity, reliance on costly and photobleachable fluorescent tags to identify the bound target, low throughput, requirement for the user to carry out probe conjugation, sedimentation, and slower reaction kinetics compared to unmodified probes [2], [3], [11], [12].

Multiplexed assay systems that employ an array of probes each identified with a molecular code, referred to as liquid or solution arrays, provide an alternative to solid support arrays and may overcome some of their disadvantages. Platforms that include DNA probes modified with individual fluorescent tags, such as used in multiplex real time PCR, are most common, but have poor multiplexing ability [13]. Probes modified with strings of fluorescent tags allow for high level multiplexing (>16,000) when detected by optical microscopy; this platform may be useful for functional genomics studies [14]. DNA barcodes attached to probes also allow solution-based hybridization, but read-out is through sequencing or chip arrays [15].

**Figure 1:**
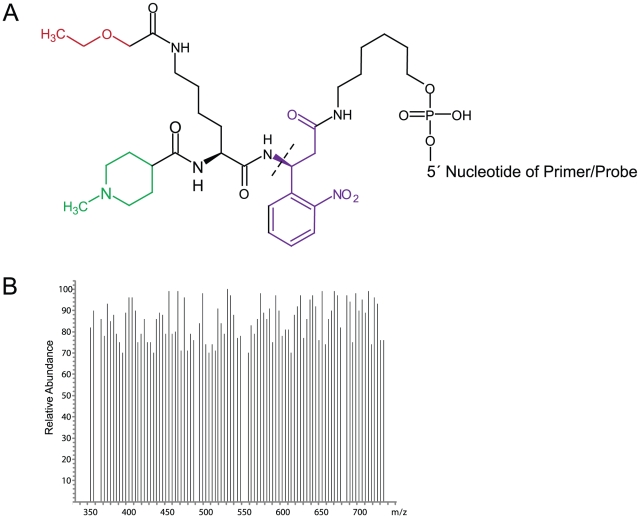
The MassCode system. A) Each MassCode reporter tag is synthesized with three functional moieties connected around a central lysine through amide bonds. The photocleavable moiety (purple) possesses a bond that is efficiently broken upon exposure to 254 nm ultraviolet light (dashed line). The sensitivity enhancer moiety (green) facilitates the formation of a stable positive ion. The molecular composition of the variable mass unit (red) provides the flexibility to synthesize hundreds of discrete tags. Currently there are 93 MassCode tags from which to choose (not shown). Despite having the same core structure, each tag possesses a unique variable mass unit and therefore each of the 93 tags has a unique total mass. B) Mass spectrum depicting the detection of positive ions of 93 MassCode tags after UV cleavage. MassCode tag masses after UV release range from 352 to 733 amu and all tags can be simultaneously detected without spectral overlap.

Arrays based on MassCode technology also employ molecular encoding of probes [16]. MassCode arrays comprise up to 93 oligonucleotides covalently modified with distinct small molecular weight tags that are soluble in aqueous solutions and released through UV exposure (Fig. 1). In essence, the organic tags provide the individual biomolecules to which they are associated with a traceable digital code that correlates to the tags' mass, and experience solution-phase hybridization kinetics. Oligonucleotides modified with MassCode tags (MCTs) have been used in simplex SNP genotyping assays then combined for multiplex detection [16]. Also, DNA primers encoded by MCTs have notably been used in multiplex PCR microbe detection assays; positive test results were verified by real time PCR [17], [18].

Often the limiting factor in multiplexing success is not the degree of encoding available, but it is in the challenge of concentrating all targets of interest (e.g. analyte amplification) in a small sample volume, an often neglected aspect of sample testing. This makes the ability to encode thousands of molecules of little practical use so far for applications such as molecular typing and diagnostics, for example, where nucleic acid amplification-based techniques dominate (e.g. PCR). Multiplex PCR is still very difficult to accomplish because there is an upper limit of multiplexing determined by an intrinsic phase transition [19], and therefore only a modest number of codes and targets (<50) have been included in differential detection assays [20]. An additional important problem to address for multiplex PCR amplification is the potential lack of specificity. As the number and concentration of primer pairs increases, so does the amplification of mispriming events [21]. Specificity and other inherent difficulties in multiplexing have therefore led to the implementation of corrective and confirmatory measures. These include employing strict computational assay design algorithms [22], [23], interrogating amplified targets with specific extenders or probes [24]–[27], and/or validating amplified target sizes [28], [29].

This report details the development of a MassCode probe liquid array system which implements measures addressing the inherent complexities and limitations of multiplexing. Of particular interest was validating multiplex PCR results by incorporating a probe extension step. As a first demonstration of the utility of the MassCode probe liquid array method a proof of principle prototype assay is also described which illustrates this platform's potential for detecting and molecular serotyping *Salmonella enterica*. Molecular serotyping of *Salmonella* isolates has been demonstrated using planar arrays [30]–[32], a microsphere suspension array [33], and multiplex PCR followed by electrophoresis [29], [34]–[37]. Robust molecular typing techniques must provide parallel acquisition of multiple data channels while still affording expedited results for many samples, all without a detrimental loss in sensitivity. The results presented in this report suggest that solution arrays based on MassCode may offer the necessary balance in performance to achieve these requirements.

## Results

### MassCode PCR

**Figure 2:**
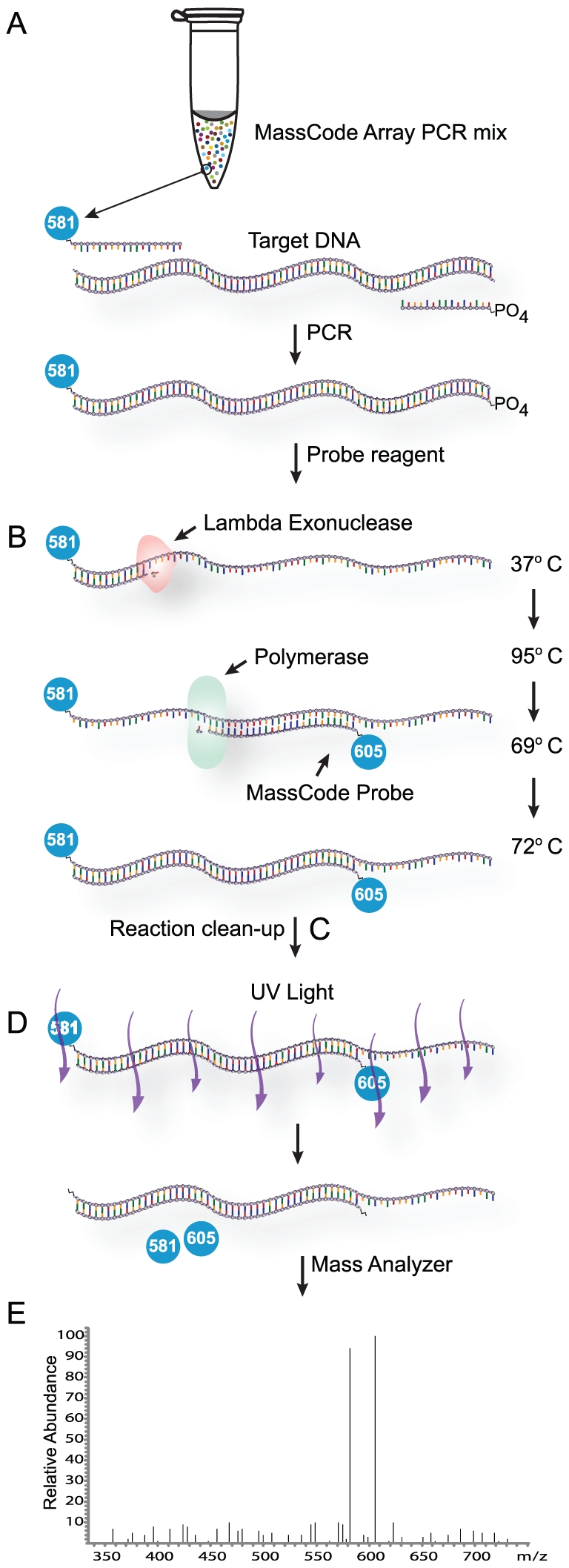
Overview of MassCode probe array assay format. A) The MassCode workflow begins with a MC-PCR reaction containing all primer pairs for each target group. One primer is labeled with MCT, the other with phosphate. Anti-sense primers are phosphate labeled if anti-sense probes are used in the ensuing step, and vice versa. B) MC-PCR products containing 5′ phosphate strands experience specific digestion of those strands at 37°C after the addition of an admixture containing lambda exonuclease. Single-stranded amplified target DNA labeled with one MCT remains, but duplex off-target DNA labeled with two MCTs and single-stranded off-target DNA labeled with one MCT also remain due to mispriming events during the multiplex PCR (not shown). Directly after digestion a second round of selection is performed during one PCR-like cycle. MassCode probes are annealed to internal sequence of the single-stranded target amplicons and serve as extension primers for DNA polymerase, the result is a double-strand single-strand segmented hybrid labeled with two unique MassCode reporters. Digestion and probing chemistry are combined into a single reagent that is added to the MC-PCR tube, making the process amenable to automation. C) Unincorporated oligonucleotides and misprimed amplified DNA less than 100 bp are removed during a reaction clean-up step. D) and E) MCTs are cleaved from the hybrids upon exposure to UV light and flowed directly into a single quadrupole mass spectrometer for detection.

MassCode multiplexing technology (Fig. 1) was used as a basis to design a new highly discriminatory nucleic acid detection system. The general scheme of the entire molecular probing process is shown (Fig. 2). The first step amplifies target DNA using multiplexed MC-PCR. In previous configurations, both forward and reverse primers in a multiplexed PCR were labeled with mass tags [17]. In the illustrated configuration, the forward primer specific for the target DNA sequence is assigned and modified with a unique MCT while the reverse primer is modified at its 5′ end with phosphate. The system is flexible such that either the forward or reverse primer can be MCT labeled as long as the other primer is phosphate labeled. Establishing which primer receives which modification is determined by the type of probe designed to be most suitable according to strict computational analysis of the target internal sequence. Anti-sense target probes require forward primers for the same target to be MCT labeled whereas sense probes require the reverse primers to be labeled with MCT and the forward primers to be labeled with phosphate. In a multi-target panel, the array of primer signatures is designed to be added to a MC-PCR master mix that is optimized to accommodate multiple simultaneous amplifications of nucleic acid targets in one reaction, if present. According to this scheme, the array mixture added to DNA extract for MC-PCR produces unique mono-labeled MCT amplicons for each present target (Fig. 2A).

### Synthesis and detection of MassCode hybrids

**Figure 3:**
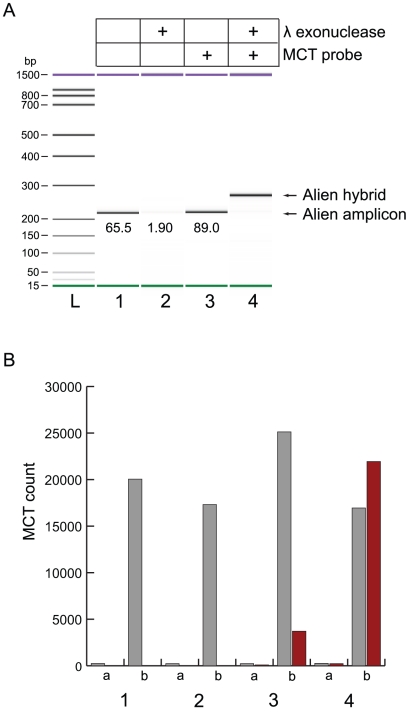
Lambda exonuclease mediates efficient MCT hybrid synthesis. A) Bioanalyzer DNA 1000 capillary electrophoresis analysis of a MC-PCR sample subjected to lambda exonuclease and/or 418 amu MCT probes during hybrid synthesis reactions. Alien DNA served as template for MC-PCR resulting in amplicons (209 bp) encoded on one end by a 352 amu MCT derived from forward priming and modified on the other end by phosphate derived from reverse priming. Aliquots of pooled amplicons served as sample input for hybrid synthesis reactions for lanes 1–4 under the conditions specified. Aliquots of a pooled MC-PCR without Alien DNA served as no template controls (NTC) for each hybrid synthesis reaction condition. The molarity in nanomolar of the Alien amplicons present at the end of the hybrid synthesis process is shown under each band. Alien hybrids possess a 103 bp double stranded segment and a single stranded segment of 106 bases. They cannot be accurately sized or quantified by CE. B) MCT detection from the molecular species formed during the hybrid synthesis reactions described in panel A. Results from NTC samples (a) were compared to those of samples containing DNA (b) for each condition tested. Gray bars, measuring forward primer binding/extension, show the response levels detected by the mass analyzer at 353 amu [M+H]^+^; red bars, measuring probe binding/extension, show the 419 amu [M+H]^+^ response levels. L, DNA ladder.

The specificity of the MassCode system was enhanced by incorporating a probe interrogation step. Added probing was initially developed at a singleplex level to demonstrate process validity. In multiple experiments it was observed that adding 20–100 pmol MCT probes to post-MC-PCR reactions to allow probe incorporation during another PCR cycle resulted in inefficient probe hybridization to target amplicons. A representative result using 20 pmol Alien control probes is shown (Fig. 3 sample 3).

A resolution aimed at conditioning PCR amplicons to permit efficient probe binding was devised by introducing lambda exonuclease into the probe admixture. This enzyme directed specific digestion of the phosphorylated strand of the duplex target amplicon that resulted from MC-PCR using a MCT forward and 5′ phosphorylated reverse primer pair (Fig. 3, samples 2 and 4). In absence of MCT probes, the molarity of undigested duplex products remaining after lambda exonuclease treatment and cycling was 1.9 nM, compared to 65.5 nM for input levels. As such, nucleotide strands resulting from reverse primer hybridization and extension were digested to 97% completion in 10 minutes at 37°C in the buffer system used. APCI-MS results show that forward primer-derived MCT target strands remained after lambda exonuclease digestion of amplified targets (Fig. 3B, sample 2), but these single stranded molecular species were not visible by CE analysis (i.e. did not bind well to the intercalating DNA dye used for CE detection).

Abundant dual MCT-labeled Alien hybrid molecules were specifically synthesized from Alien post-MC-PCR products when both lambda exonuclease and Alien-specific probes were present in the admixture. Definitive qualitative determination of their formation was obtained through both CE and MS results (Fig. 3, sample 4). Optimization of the probe process showed that using 7 pmol of MCT probes per target in a multiplexed assay led to results that did not vary significantly from using 20 pmol, and that using lower amounts helped reduce background and cost of the reaction (data not shown). Extension of the probe increases the specificity of this process even further since polymerases are sensitive to mismatched bases at the 3′ of oligonucleotides.

### Identification of MassCode hybrids

A very reproducible phenomenon was observed whereby MassCode hybrids which have significant ssDNA segments migrated through the gel matrix of capillaries at a slower rate than fully dsDNA; accordingly, in these instances, CE analysis resulted in a new DNA band with an apparent larger molecular weight relative to its parent double stranded PCR product, despite the hybrid's smaller theoretical size. For example, dual-labeled MassCode Alien hybrids formed after conditioning 209 bp Alien MC-PCR products were predicted to possess a double stranded segment of 103 bp and a single stranded segment of 106 bases (Table 1), but these hybrids were observed to migrate similarly to molecules with 271 bp of DNA (Fig. 3).

**Table 1:** Primer and probe signatures used in the 14plex *Salmonella* MassCode probe liquid array.

Marker	Locus	Primer Type	5′ Mod*	Nucleotide Sequence	Amplicon	Hybrid size
					(bp)	(ds/ss)‡
pan *S. enterica*	lysR	F	356	CTA CAT TCC TTC CTG ATA TTG T	207	
		R	P	ATC CAG CAT TAT TTT GTT AGC		
		Probe	422	TGC TGA CTT AAT GAT GGC TGA GTG		89/118
O-group B	abe	F	366	AAT TGA TAA CTC CTC GAC TAA T	164	
		R	P	CTT CCG GCT TTA TTG GTA A		
		Probe	426	GGA TTT CAG TTG TCG CAA TCA CTC		116/48
O-group C1	SCH-2097	F	370	GGT AAT ATA GCC ATG TCA GTT	218	
		R	P	GGA AAG GGA ATA GAA GAA TTT ATC		
		Probe	430	GCT CTG CCT TGA TTG GTT ATG TTC		154/64
O-group C2	rfbJ	F	374	TGA AGG TCA GGG ATA CTA TT	202	
		R	P	AGC ATA AAC GCC ATC AAT		
		Probe	434	GCA ATT AGC AAC AAG CCT TCA ACC		159/43
O-group D1-A	prt	F	378	ACT GGT AAA CTT ATC GTC TC	204	
		R	P	GTA TTA GAA TCT ATC TCA TCA CTT G		
		Probe	438	GAA CAT CAC TGC CAC CAA ATA CGA		158/46
O-group E1	wzx	F	386	GGA ATA AGT AAA GTC AGT TCA AG	219	
		R	P	CAG CAC CAT ATA CTT TAA CAA A		
		Probe	442	TCC TAT CTG AGA CCC AAG AGC AAC		146/74
O-group G	wfbI	F	390	TCA GAG AAG CAA TAA TAC AAC T	174	
		R	P	CGA ACA TCA TCA GAG AAG AT		
		Probe	446	TTT GTT TAC CTC GCT CAC GCT CTA		146/28
Typhimurium	STM0893	F	394	CAG CGT TTC TTT ATT AGG AG	220	
		R	P	TGG GTT TTG TGG AAT GTA		
		Probe	450	ACG GGC AGC AAA CTG AAA TAA TCC		196/24
Agona	SeAg-B2803	F	398	TTA TGA CGC TCA CTT ACT G	220	
		R	P	TGT TTG ATT ACC TGG ATG AA		
		Probe	486	TGG CAC CTT ATG GCA TCA ATC ATT		199/21
Enteritidis	SEN1383	F	402	AAC TCT TCG GGT TTA ACT C	153	
		R	P	GCG AGA CCT CAA ACT TAC		
		Probe	458	TGG GCT TTG AGA CAC AAT CTA CCG		112/41
Dublin	SeD_B0058	F	406	TGT AAC TAA TGG TCA CAG AAT	214	
		R	P	TGT AGT TCA CCG TAT AGA ATC		
		Probe	462	CCA GAA GAG ACG GTG TTG ACA AG		131/83
Typhi	STY2074	F	410	CGG AGA AAC AAC ATC ACT	150	
		R	P	GAA GGC AGG TCA TTT ATC A		
		Probe	466	CAT TGA GAC GGT GAT GAC GCT GAA		110/41
Paratyphi A	SPA2473	F	478	ACC CGA TGT AAA ATC ACT C	213	
		R	P	GGG AAA TAC AGT AGT TTG GT		
		Probe	470	GTC CCG TCA GTT ATA ATG ATG CGG		191/22
Alien	Alien	F	352	AGT TTG CAA GTG TTA GCT	209	
		R	P	CTA GTT TAT CCA CTC CGA TAG		
		Probe	418	GTT GAC TGC CGT AAA CTT GGG T		103/106

‡ ds, double stranded segment length in base pairs; ss, single stranded segment length in bases.

*Modification with a number denotes MassCode mass; P, phosphate.

**Figure 4:**
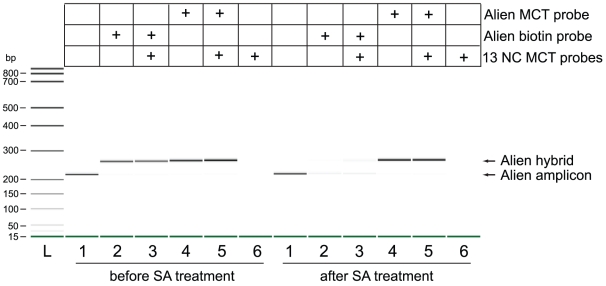
Identification of MCT hybrid species formed from specific probe hybridization in simplex and multiplex mode. Alien post-MC-PCR products (209 bp) were pooled and aliquotted into 6 samples. Hybrid synthesis reactions were carried out on samples 2–6 after adding probe admixture containing either Alien MCT probes only, Alien biotin probes only, a multiplex mixture of 13 MCT probes non-complementary to Alien target (13 NC MCT probes)(Table 1), or both the 13 NC MCT probe mix and an Alien probe. NTC post-MC-PCR reactions, run using Alien primers but no template, were subjected to all 5 hybrid synthesis conditions but resulted in no DNA species (data not shown). All samples were cleaned prior to incubation with SA-coated magnetic beads. The flow through of each sample was precipitated by ethanol and the eluate was analyzed in a DNA 1000 chip by the Bioanalyzer 2100.

To help confirm hybrids were being correctly synthesized, the MCT 418 reporter on the Alien probe was replaced with a biotin and all molecular species that incorporated the biotin probe during the probe cycle were captured by streptavidin (SA) coated magnetic beads. The molecular species that remained were compared to those that were formed from using MCT probes (Fig. 4). Hybrid synthesis reactions using Alien MCT probes only, Alien biotin probes only, Alien biotin probes in a background of thirteen other MCT probes, or Alien MCT probes in a background of thirteen other MCT probes all resulted in a visible DNA band of approximately 271 bp. Upon treating these samples with SA beads, the 271 bp bands produced from samples which had utilized the Alien biotin probes were captured. Hybrids formed from MCT probes were unaffected by treatment with SA beads. Since the probe sequence was identical in all reactions, the results showed that the 271 bp molecular species was formed from specific Alien probe binding to single stranded Alien post-MC-PCR products.

### Design of a MassCode probe liquid array

**Figure 5:**
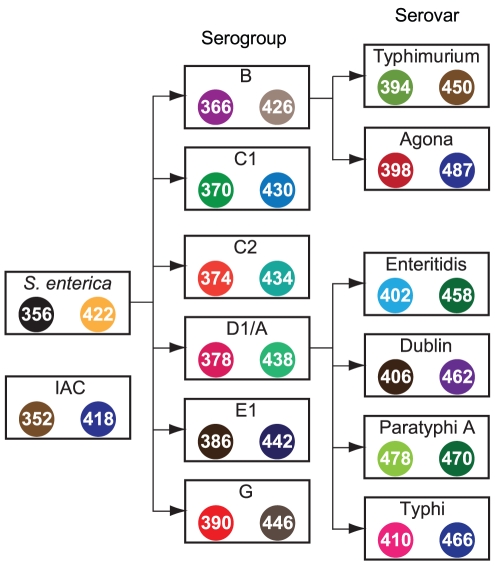
Hierarchy of the 14plex *Salmonella* MassCode probe liquid array design. The serogroup/serovar lineages are based on Kauffmann-White formulae. Each category (boxed) is detected by one genetic marker (Table 1). In this particular design, the detection of each marker is correlated with two distinct MCTs. The left and right MCTs represent the modification of the forward primers and probes, respectively; both MCTs must be detected to identify each marker. The *S. enterica* locus is a pan locus that detects all *Salmonella enterica enterica*. To accept a serogroup or serovar subtyping result as valid, multiple markers must be simultaneously detected according to the logical direction of the arrow flow.

The methods described were devised to enable the development of an assay concept that could be scaled up to achieve high-throughput reliable multiplexed analysis of samples. To this end, a *Salmonella* molecular serotyping assay was developed to serve as a vehicle through which MassCode probe multiplexing technology could be demonstrated. The proof of principle 14plex *Salmonella* MassCode array presented uses a panel of six genetic markers within the *rfb* gene cluster to unambiguously identify salmonellae belonging to common serogroups B, C1, C2, D1/A, E1, and G [33]. Comparative genomic analysis identified another six candidate target gene markers that distinguish serovars Typhimurium, Enteritidis, Agona, Paratyphi A, Typhi and Dublin from other salmonellae serovars and all other non-related taxa with sequence availability in NCBI (Table 1). A pan *S. enterica* marker was also included to determine presence/absence of salmonellae within different taxa. The assay is organized in a hierarchical fashion whereby in order to positively subtype an isolate that is detectable using these panel markers, multiple loci that designate the serovar and/or serogroup, must be detected in parallel with the pan *S. enterica* locus and IAC (Fig. 5). If no *Salmonella* loci are detected, then the IAC must be positive to suggest no false negative.

Analysis of the genetic markers resulted in the design of an array of 14 dually-labeled primer/probe signatures chosen for their capacity to specifically bind and amplify the 14 markers. The array employs 42 oligonucleotides, of which there are 14 MCT forward primers each paired with one of 14 5′P reverse primers for MC-PCR, and 14 MCT internal anti-sense probes, each of which is matched with a primer pair (Table 1). To increase probability that the 42 primers and probes would be compatible since they are present in the same reaction, the signatures were designed using multiplex PCR design software such that each was predicted to not cross-hybridize with one another or itself based on various free energy calculations, such that the average primer T_m_ was 61°C (standard deviation = 0.3°C), and such that the average probe T_m_ was 69.8°C (standard deviation = 0.8°C).

Each dually-labeled MCT signature was first tested against their respective DNA targets in singleplex MC-PCR reactions and it was confirmed that each primer/probe set was able to identify its intended target through the synthesis and detection of its correlative hybrids (data not shown). To assemble the multiplex array, individual MCT signatures were added to a growing mixture one at a time and demonstrated to work during each iterative cycle until all fourteen signatures were added.

### Application of MassCode arrays allows multiplexed genetic analysis of samples

**Figure 6:**
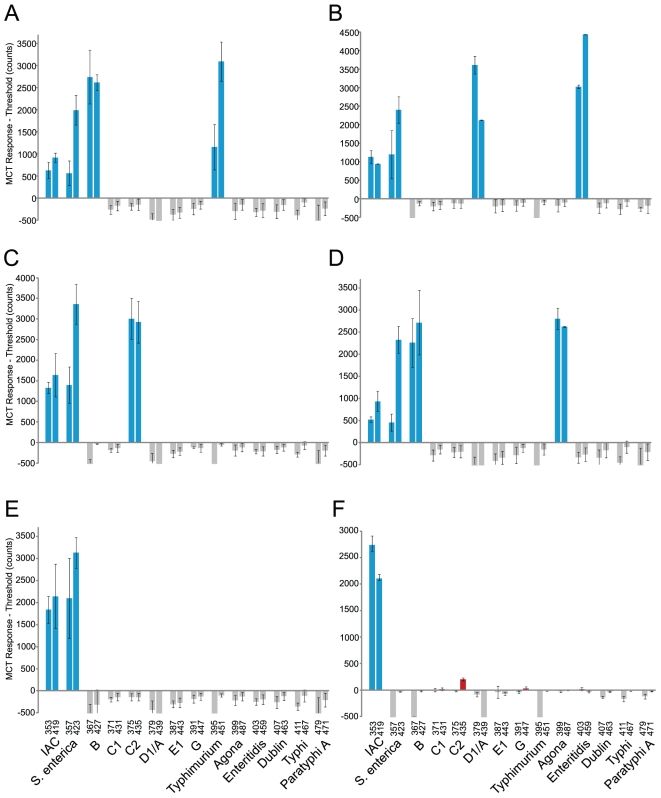
Detection and subtyping *Salmonella* isolates using a prototype MassCode probe liquid array assay. A) *Salmonella enterica* Typhimurium SGSC1412 (LT2) (serogroup B), B) *Salmonella enterica* Enteritidis SGSC4901 (serogroup D1), C) *Salmonella enterica* Newport SGSC2493 (serogroup C2), D) *Salmonella enterica* Agona SGSC2458 (serogroup B), E) *Salmonella enterica* Rubislaw SGSC2511 (serogroup F (O:11)), and F) *E. coli* O157:H7 EDL933 gDNA were analyzed by the MassCode array in parallel with NTC samples to obtain serovar-specific (A,B,D), serogroup-specific (C), *Salmonella*-present (E), or *Salmonella*-absent MCT profiles. The 28 masses monitored (x-axis) represent the [M+H]^+^ value of the 28 MCTs that make up the assay. The left and right mass above each target represent the MCT associated with the forward primer and probe used to identify that target respectively. The response for each ion is displayed as the average of three biological replicates individually tested in three different experiments over a thirteen day period, minus the threshold for that particular MCT. Error bars represent one standard deviation of replicates. MCT responses that were positive (i.e. above zero) and were expected to be positive are shown in blue. MCT responses that were negative (i.e. below zero) and were expected to be negative are shown in gray. MCT responses that were positive but were expected to be negative are shown in red ([M+H]^+^ 431, 435, 447, and 403 of panel D). No responses were negative that were expected to be positive. IAC, internal amplification control.

The utility of the MassCode probe liquid array analysis system to detect and subtype bacteria was demonstrated by using the 14plex *Salmonella* array to test DNA samples from various *Salmonella* isolates and phylogenetically related bacteria. All samples were compared to NTC reactions of the full multiplex panel run in parallel to determine the positive (presence) or negative (absence) call status of each of the 28 MCTs in the processed array (see *Materials and Methods*). Testing 100 pg DNA from *Salmonella enterica* subspecies *enterica* serovar Typhimurium strain LT2, for example, resulted in eight positive MCT responses that thus composed the MCT profile 352_418_356_422_366_426_394_450 (Fig. 6A). This profile correlated to the amplification, hybrid synthesis, and detection of markers IAC, pan *S. enterica*, serogroup B, and serovar Typhimurium. This was the expected profile for Typhimurium based on its hierarchy and MCT assignments (Fig. 5). All other MCT signals monitored were negative with respect to negative controls. The six positive MCT ion signals originating from the synthesis and detection of three *Salmonella*-specific hybrids allowed accurate identification of this Typhimurium isolate to its genus, species, subspecies, serogroup, and serovar level from a single test. Rarer isolates of salmonellae do not result in a negative result in this assay. Serovar Rubislaw, for example, is not a member of the common serogroups or serovars subtyped by this particular array, but it was detected by virtue of its pan *S. enterica* locus (Fig. 6E). This locus grants this array the ability to detect uncommon isolates so that at minimum an alert is placed on such samples during surveillance.

The specificity of the assay was further demonstrated by testing against an exclusivity panel. A typical negative result, that from testing *E. coli* O157:H7 DNA, is shown (Fig. 6F). Four unexpected MCT positive responses were obtained, resulting in the profile 352_418_430_434_446_402. This profile did not correlate with any logical combination of MCTs for any *Salmonella* serogroup/serovar outlined in Figure 5, and only the internal control marker was detected. These low level isolated MCT positives did not result in false-positive detection of targets in a dual label system. Moreover, they were not due to amplification of any target since DNA amplicons greater than 100 bp were absent (data not shown). Instead, they are due to slightly increased background levels in negative samples for those particular positive MCTs compared to the background levels of the same MCTs in NTC controls, resulting in responses that are above but close to the threshold level for that particular MCT (Fig. 6F).

### Correlation of replicates and sensitivity of the *Salmonella* multiplexed typing assay

**Figure 7:**
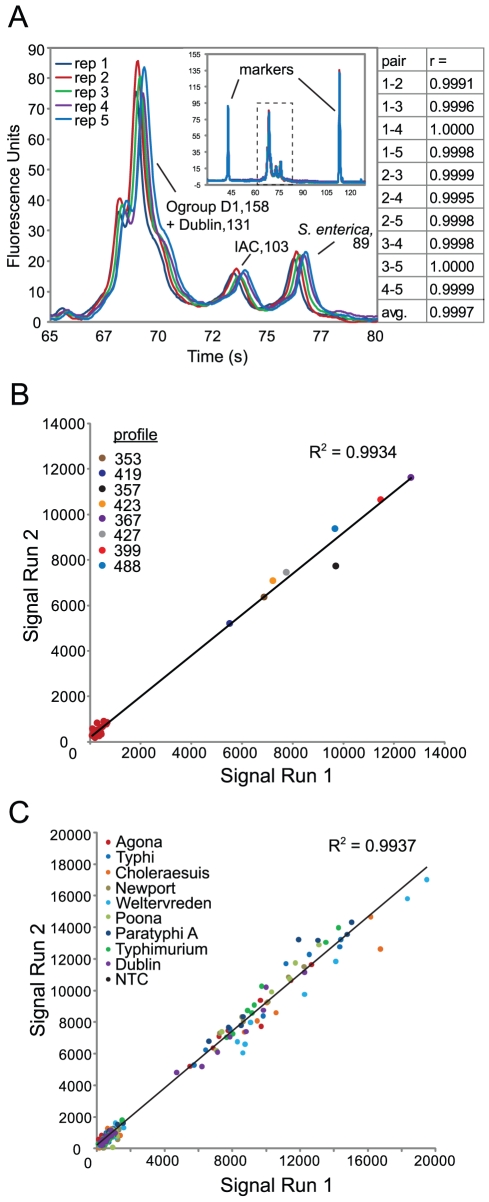
Reproducibility of the MassCode probe array platform. A) Zoom of electropherogram of hybrid duplex DNA fluorescence versus separation time for five technical replicates spiked with 100 pg serovar Dublin gDNA and assayed by the *Salmonella* array. The four hybrids detected by CE analysis are annotated with the length (bp) of duplex DNA for each hybrid. The integrated peak areas for three resolved peaks were used to calculate the correlation coefficient (r) for all pairwise comparisons of technical replicates. The inset shows the entire electropherogram outside of the boxed zoomed region, no off target species can be seen. B) Scatter plot for all 28 MCT ion counts monitored in the assay after spiking technical replicates with 100 pg serovar Agona gDNA. Negative MCTs form a cluster near the origin and are clearly separated from 8 positive MCTs that report four hybrids with an Agona-specific profile. C) Scatter plot of 280 MCT ion counts combined from technical replicates of ten different spikes at 100 pg DNA each. Each colored dot represents 1 of 28 MCTs associated with each designated salmonellae.

Overall, the MassCode system exhibited good inter-assay correlation, which was examined over a 13 day period. Samples returned the same final qualitative result whether tested on day 1, 7, or 13; though, the average coefficient of variation for quantified responses was 34% (Fig. 6). The reproducibility of the MassCode probe platform in synthesizing mass encoded hybrids was examined using serovar Dublin DNA. CE peak area measurements of Dublin hybrid sets generated from replicate tests (*n* = 5) were very reproducible. The average correlation coefficient from each pairwise combination was 0.9997±0.0003 (Fig. 7A). Intra-assay variability in APCI-MS detection of MassCode hybrids was also examined. The counts from all 28 MCT ions monitored from two independent tests of serovar Agona DNA run in parallel are shown (Fig. 7B). The R^2^ value of a linear fit to this data was 0.9934 and represents a strong correlation between the replicates. Furthermore, analysis from 280 MCT counts acquired from testing replicates of ten different spiked samples also demonstrated that the MassCode system is reproducible: a linear fit to the data resulted in an average correlation of determination of 0.9937±0.0024 (Fig. 7C).

The analytical sensitivity of the *Salmonella* MassCode array was investigated to determine any variation in sensitivity as the numbers of target loci in one reaction increase. The 14plex array was applied to DNA dilution series from serovars Typhimurium, Montevideo, and Rubislaw that produced four, three, or two dually-labeled MCT hybrids, respectively, including the IAC. Six log dilutions of DNA were prepared so that an aliquot of each dilution to seed the MC-PCR reaction contained between 3 to 300,000 *Salmonella* spp. genome equivalents. Simultaneous amplification, hybrid synthesis, and correct detection of 4 MCTs from the pan *S. enterica* and IAC targets in the 14plex were observed for Rubislaw down to a concentration of 300 genome equivalents, (Table 2). For correct subtyping of samples requiring simultaneous identification of three or four loci per run an input of at least 3,000 genome equivalents (i.e. 3,000 copies of each target) was needed based on the primer/probe signature performance for Typhimurium and C1 targets. The ten other serogroup and serovar signatures performed similarly (data not shown). Only the internal control marker could be detected for samples with starting copy numbers of 30 or 300 and below, dependent upon whether two or more loci, respectively, would have been required for detection.

**Table 2:** Detection results from dilution series of *Salmonella* DNA containing multiple target loci‡.

			Assay markers and their corresponding MCT ions*									
			IAC		*S. enterica*		B		C1		Typhimurium	
Isolate	Loci	Copies∧	353	419	357	423	367	427	371	431	395	451
Typhimurium	4	3×10^5^	+	++	++	+++	+++	+++	−	−	+++	+++
		3×10^4^	++	++	++	+++	+++	+++	−	−	++	+++
		3×10^3^	++	++	+	+++	+	++	−	−	++	++
		3×10^2^	+++	+++	−	+	−	−	+	−	−	−
		3×10^1^	+++	+++	−	−	−	+	−	−	−	−
		3×10^0^	+++	+++	−	−	−	−	+	−	−	−
		0	+++	+++	−	+	−	−	−	−	−	−
Montevideo	3	3×10^5^	++	++	++	+++	−	−	+++	+++	−	−
		3×10^4^	++	++	++	+++	−	−	++	+++	−	−
		3×10^3^	+++	+++	+	++	−	−	++	+++	−	−
		3×10^2^	+++	+++	−	−	−	−	−	+	−	−
		3×10^1^	+++	+++	−	−	+	−	−	−	−	−
		3×10^0^	+++	+++	−	−	−	−	−	−	−	−
		0	+++	+++	−	+	−	−	−	+	−	−
Rubislaw	2	3×10^5^	+++	+++	++	+++	−	−	−	−	−	−
		3×10^4^	+++	+++	+++	+++	−	−	−	−	−	−
		3×10^3^	+++	+++	++	+++	−	−	−	−	−	−
		3×10^2^	+++	+++	+	+	−	−	−	−	−	−
		3×10^1^	+++	+++	−	−	−	−	+	−	−	−
		3×10^0^	+++	+++	−	−	−	−	+	−	−	−
		0	+++	+++	−	−	−	+	−	−	−	+

‡ The results are presented as a minus sign for responses of ≤0 counts over threshold, a plus sign for responses of >0 but ≤500 counts over threshold, two plus signs for responses of >500 but ≤1,500 counts over threshold, and three plus signs for responses of >1,500 counts over threshold. The threshold for each MCT ion was calculated from 8 NTC samples. Responses are calculated from the average of five replicates.

*All 28 MCTs assigned to the fourteen primer/probe signatures were included and monitored in the assay, but for clarity only responses from positive targets are shown. The other 9 markers for all dilutions were called negative after analyzing the responses from both MCTs of each marker. The left MCT ion of the target pair monitors incorporated F primer; the right MCT ion monitors incorporated probe.

∧ Copy number for each *Salmonella*-specific target loci. BLAST analysis indicated that gene targets were single copy per genome. The dilution of stock DNA to obtain the starting concentration (3×10^5^) for making the 6 log dilutions was calculated based on a genome size of 4.8 megabases.

## Discussion

A rapid multi-loci gene analysis system was developed that achieved high sensitivity, was reproducible, and provided three-tier sequence-specific interrogation of DNA targets. Multiple targets were simultaneously encoded with unique MassCode tags incorporated from labeled primers and probes using processes modified from standard PCR. Several key measures were taken to reconcile the complexities and normally lower performance of multiplex PCR [19], [21], [38]. Computational analysis software was employed to design the assay to increase probability of primer set compatibility and to help curtail high background. Crucially, the algorithm was also capable of designing for even, size-unbiased amplification and very similar melting temperatures. Software for multiplex taxa specific assay design tailored specifically to MassCode liquid array technology is in development.

Consistent with other nucleic acid tests, the application of MassCode arrays requires prior knowledge of nucleic acid target sequences in order to design precise oligonucleotides. However, MassCode primers have previously been successfully employed which were not strictly complementary to their target. For example, by designing degenerate genus-wide primers a novel rhinovirus genotype was discovered using MassCode multiplexing [39], [40]. The application of multiplex MC-PCR has been concentrated in microbial genotyping where unique targets were identified for differential detection. So far, multiplex MC-PCR has not been tested for its ability to differentiate targets with a higher order of identity (e.g. alleles that vary according to single nucleotide polymorphisms). But, the PrimerPlex software used to design the *Salmonella* assay is capable of designing multiplex-compatible allele specific probes for SNP genotyping assays. This feature provides at least a theoretical feasibility that the MassCode system could be used for other applications such as pharmacogenomics and clinical research.

Despite great improvement through the use of pairwise primer compatibility algorithms, a certain level of incompatibility of primers is unavoidable. Due to all sets being present at necessarily high concentrations, most all primers are implicated in the formation of heterogeneous exponentially-amplified off-target species, a problem that increases quadratically as the level of plexing rises [19]. Also, in complex matrices (e.g. food and environmental samples) false positive results are observed more frequently since non-specific amplification readily occurs [41]. To address these potential issues in the MassCode system the specificity of the MC-PCR was validated, in the same tube, by performing a one-step multiplexed quasi-nested probe reaction.

To mediate probing, lambda exonuclease was employed to specifically digest one strand of each target amplicon in order to bypass the favorable kinetics of strand re-annealing. Lambda exonuclease is a very efficient and processive enzyme motor that catalyzes the removal of 12 mononucleotides per second in the 5′ to 3′ direction of, preferentially, phosphorylated duplex DNA [42]. Amplifying relatively short amplicons allowed rapid completion of nuclease activity. Digestion was very robust and proved essential to achieve greater probing sensitivity. Through optimization it was found that efficient MCT hybrid synthesis could be mediated by adding to an entire post-MC-PCR reaction a single 25 µl mixture that included MCT probes, lambda exonuclease, and DNA polymerase as its temperature-directed ‘active ingredients’. Relative to hybrid synthesis reactions where the PCR products were not conditioned prior to probing, lambda exonuclease treated samples normally showed at the very least a 5 to 10 fold increase in probe MCT detection, but several log increases were common.

We showed that using a MassCode-based approach for genetic analysis provided a feasible means with which to discriminate salmonellae within the same subspecies in one test. The assay is proof of principle and the serovars chosen for subtyping are based on six of the thirteen to date *Salmonella enterica* subspecies *enterica* reference genomes available from NCBI. Unfortunately, these references genomes do not correspond precisely to those serovars most often implicated in Salmonellosis outbreaks. Still, the hierarchical format used in the *Salmonella* MassCode array was able to indicate samples contaminated with *Salmonella* isolates less frequently encountered in food, while subtyping to the serovar level samples contaminated with some salmonellae more frequently encountered. Future versions of a subtyping MassCode array do not necessarily have to be hierarchical. For example, the pan *S. enterica* and serogroup targets of this assay could be replaced and more serovar-specific targets added. In such case, an even more sensitive *Salmonella* subtyping assay that could detect 300 genome equivalents can be envisioned, since results would be expected to be similar to those obtained from testing serovar Rubislaw in this report.

This study did not assess the potential interference of the MassCode system from food matrices after overnight primary enrichment of *Salmonella*-contaminated foodstuffs since we primarily focused on creating a molecular methodology and integrating it with the MassCode detection system. However, rapid methods based on PCR to detect foodborne pathogens do succumb to food matrix effects, and therefore the analytical sensitivity reported here may very well decrease if PCR inhibitors are still present after DNA extraction. Testing of samples obtained from Salmonellosis outbreaks will be required in order to definitively determine whether or not simultaneous foodborne pathogen detection and subtyping is a suitable application for the MassCode probe liquid array approach. Nevertheless, if detection of real world *Salmonella* samples is more efficiently achieved using other methods, a high-throughput multiplexed genetic profiling method, like the MassCode probe liquid array, would still be highly desirable for molecular serotyping of detected isolates since current methods take an additional 4–5 days. Detection and serotyping for *Salmonella* characterization traditionally are segregated into multiple tests, and the latter process starts with a pure bacteria colony, as was done in this report.

The MassCode platform is high-throughput capable and therefore could provide a rapid screening of many samples when necessary. A MassCode array only requires 1 well per sample, which for the *Salmonella* assay reported equates to 14 tests per well and 1,344 tests per 96-well plate. The automated liquid sampler used for injection holds two 96-well plates and the mass spectrometer analyzes one sample per minute, 96 samples in 1.6 h, or up to 192 samples in 3.2 h. The PCR and probing sample preparation and clean-up procedure upstream of detection consists of pipetting steps and a bind/wash/elute workflow that are automatable. Without automation, a user could process samples prior to detection analysis in two 96-well plates simultaneously from beginning to end in 3.5 h (1.75 h hands-on time, 1.75 h temperature cycling). Starting with DNA, therefore, total time to results would be about 7 h for 192 samples and about 5 h for 96 samples.

The overall performance of the MassCode analysis system correlates well with existing multiplex technologies that have been used for pathogen detection and typing, but advantages and disadvantages can be noted. Some advantages are the following: (1) The MassCode system employs primers, probes, targets and codes that are all in homogeneous solution during target hybridization steps rather than bound to a surface or interacting with a solid/liquid interface as with suspension arrays and microarrays [30]–[33]. (2) Real-time or electrophoretic-based nucleic acid amplification and detection assays also can perform solution phase interrogation of target DNA, but the assays are not highly multiplexed or high-throughput, respectively. (3) Each MassCode oligonucleotide solution arrives HPLC purified and pre-coupled to its unique mass tag identifier. On the other hand, microsphere suspension array-based protocols for bacterial detection and molecular typing, for example, require the user to couple probes to beads, purify, and provide quality control and validation of the coupling and final suspension for each probe/bead set prior to use [4], [33]. (4) Results from each MassCode test have quality assurance measures built in since positive target identification is concluded only when two correlative MassCode signals are positive for that target. This is in contrast to some microsphere arrays that require a single signal (e.g. phycoerythrin) to determine whether a target is bound or unbound to bead-coupled probes [25]. Other single signal assays have built in test triplicates for call-making assurance [31], [43]. (5) MassCode-based assays allow flexibility in their design. Unlike for particle suspension arrays, mass tag labels have been used successfully on both primers and probes. Also, oligonucleotides are chosen for their unbiased amplification and multiplexing suitability instead of their ability to synthesize amplicons that must be separated by size like in electrophoretic systems.

The major disadvantage of the current versions of MassCode is that, unlike in real time PCR, MassCode assays do not take place in closed tube systems. Post-PCR steps in the MassCode protocol thus may increase contamination risk. However, the state of the art of all high density and moderately multiplexed detection platforms so far reported include open tube processing of samples, and implementing appropriate control measures are suggested [44]. Post-PCR steps also add to hands-on time. Future versions of analytical systems based on MassCode may include automated purification steps, or new and automated purification methods, that may decrease contamination risk.

Presently, the instrumentation required for MassCode analysis is modular and the mass spectrometer must be in vacuum, but the modules are connectedly seamlessly, and future versions may be completely integrated. Furthermore, mass spectrometry is gaining acceptance for environmental and clinical microbiology [45]. The MassCode system could help facilitate a transition since it employs a relatively straightforward benchtop mass spectrometer. Also, any procedural complexity around liquid handling and mass spectrometry is largely reduced since samples are subjected to automated injections, and some instrument maintenance procedures are also automated. MassCode software provides instrument control and automated data analysis through a user-friendly GUI. The MassCode analysis system is not restricted to pathogen identification. One could envision the MassCode approach applied to molecular diagnostic fingerprinting, GMO testing, adulteration, and allergen testing, to name a few.

## Materials and Methods

### MassCode PCR

MassCode reporter tags (Agilent Technologies) are conjugated to oligonucleotides through a 6-amino-1-hexanol linker attached to the 5′ terminal phosphate of DNA primers and then HPLC purified (Eurofin MWG Operon). PCR reactions were performed in 25 µl total volume (12.5 µl 2× Brilliant Multiplex Master Mix (Agilent Technologies), 300 nM each primer, 1 µl genomic DNA sample, and nuclease-free water. SureStart Taq DNA polymerase in the master mix provides high-specificity hot-start and is activated after 10 min at 95°C. Amplification was performed for 30 to 36 cycles, each at 95°C for 30 s, 59°C for 30 s, and 72°C for 30 s. Amplicons were designed to be between 140 to 220 bp. The MassCode liquid array system can be run in either single tube or 96-well plate formats.

### MassCode hybrid synthesis reaction and clean-up

An admixture (25 µl) containing lambda exonuclease (7.5 U, New England BioLabs), *Paq*5000 polymerase (1.25 U, Agilent Technologies), MassCode probes (150 nM each), *Paq*5000 10× hot start buffer (5.25 µl), and nuclease-free water was added directly into the entire post-PCR sample and placed back on the thermal cycler. A set of temperature dependant sequential reactions took place during one more cycle. Lambda exonuclease digested the reverse strand of double stranded PCR amplicons at 37°C for 10 min, lambda exonuclease was inactivated and polymerase activated at 95°C for 2 min, MassCode probes annealed and extended at 69°C for 1 min to form the dual-labeled MassCode hybrids, and hybrid extension was completed at 72°C for 3 min. Samples were purified using the silica-based StrataPrep or StrataPrep 96 PCR purification kit (Agilent Technologies). The manufacturer's instructions were followed with the exception that an equal volume of 70% ethanol was added to the binding buffer used in step one of the kit's instructions. Samples were eluted in 60 µl nuclease-free water.

Oligonucleotide MassCode probes (24 bp) for each amplicon target were assigned and modified with MCTs that were unique among the other modified probe and forward primer signatures. Probe sequences represented the reverse compliment to an amplicon target sequence of the remaining strand and were designed against internal sequence between the primer pairs for that target. The system worked best with a distance greater than 100 bases between the 3′ end nucleotide of a correctly hybridized probe and the 5′ end of the remaining forward strand of the amplicon target. MassCode probes were designed to exhibit an average melting temperature of about 9°C higher than that of its target's primer set.

### MassCode tag detection and analysis

Purified samples were placed into a 96-well plate holder or single vial holder of an Agilent 1200 Series HiP-ALS (High Performance Automated Liquid Sampler, G1367B) and sequentially injected by 0.8 ml/min flow from an Agilent 1200 Series Isocratic pump (G1310A). Samples were flowed through an inline UV photolysis unit (Aura Inc.) to cleave MCT reporters from their respective DNA molecules. Detection took place in positive single ion monitoring mode by atmospheric pressure chemical ionization mass spectrometry (APCI-MS) using a benchtop Agilent 6100 Series Single Quadrupole MS (G6120B). Instrument control and automated data analysis were performed with MassCode analysis software (Agilent Technologies).

Positive or negative call status for each MCT was determined by comparing the signal intensity of each individual MCT from a test sample to the baseline signal of the same MCT from no template control (NTC) samples run in parallel. The average of a particular MCT signal calculated over multiple NTC samples formed the baseline signal for that MCT. These baseline responses were then used to establish threshold values for individual tags. The threshold was defined as 3.3 times the standard deviation above the baseline signal. The number of NTC reactions used to determine the threshold varied in each experiment and is indicated in the text. In some instances, the threshold of each tag was subtracted from the response of that particular tag in the sample. A positive call was thus applied to sample MCT signals that were greater than zero, and a negative call was applied to sample MCT signals that were less than or equal to zero. In the dual label system presented here, detection of a target (marker) must be the result of making two positive calls, one for each of the MCTs correlated with that target locus. If only one of the two target MCTs is positive, it is considered a non-specific response and the target is considered absent. False positive target detection results only when both MCTs associated with a target are positive, but the target DNA was truly absent.

### Salmonella isolates and molecular typing assay

*Salmonella* isolates were purchased from the Salmonella Genetic Stock Center (University of Calgary) and grown in 8.0 ml BHI (Teknova) overnight in a shaking 37°C incubator prior to removing 1.0 ml of the culture for genomic DNA extraction (Qiagen DNAeasy). DNA was quantified by spectrophotometric analysis with the NanoDrop 1000 (Thermo Scientific). A DNA sequence with no significant homology to any known sequences named Alien (Agilent Technologies) served as a PCR internal amplification control (IAC) when added to test samples. Serovar-specific targets were found through computational comparative genomic analysis using BLAST. Segments of each serovar genome were sequentially searched until a specific locus was identified that did not show homology with other salmonellae or non-salmonellae genomes in NCBI. PrimerPlex 2.0 (Premier Biosoft International) was used for multiplex PCR signature design. The resultant signatures are shown (Table 1).
